# Environmental and demographic drivers of male mating success vary across sequential reproductive episodes in a polygynous breeder

**DOI:** 10.1002/ece3.5066

**Published:** 2019-04-10

**Authors:** Jeffrey A. Manning, Philip D. McLoughlin

**Affiliations:** ^1^ Department of Biology University of Saskatchewan Saskatoon Saskatchewan Canada; ^2^ Present address: School of the Environment Washington State University Pullman Washington

**Keywords:** environmental gradient, *Equus ferus caballus*, reproductive episode, resource availability, sex ratio, sexual selection

## Abstract

Ecological and social factors underpinning the inequality of male mating success in animal societies can be related to sex ratio, sexual conflict between breeders, effects of nonbreeders, resource dispersion, climatic conditions, and the various sequential stages of mating competition that constitute the sexual selection process. Here, we conducted an individual‐based study to investigate how local resource availability and demography interact with annual climate conditions to determine the degree of male mating inequality, and thus opportunity for sexual selection across two sequential reproductive episodes (harem and subsequent mate acquisition) in a naturally regulated (feral) horse population in Sable Island National Park Preserve, Canada. Using a 5‐year, spatially explicit, mark‐resight dataset and hierarchical mixed‐effects linear modeling, we evaluated the influence of adult sex ratio (ASR) on mating success and then tested for effects of freshwater availability, density, unpaired male abundance, and precipitation during each breeding season. Unpaired male abundance, freshwater availability, and ASR differed in their effects on male mating success according to year and selection episode. Opportunity for sexual selection in males associated with harem acquisition increased with ASR, and unpaired male abundance further explained weather‐related interannual variation after accounting for ASR. In contrast, once a harem was secured, ASR had little effect on male mating inequality in regard to acquiring additional females, while interannual variation in mating inequality increased with decreasing freshwater availability. Our findings show that local demography, resource availability, and weather effect opportunity for sexual selection in males differently depending on selection episode, and can attenuate or accentuate effects of ASR.

## 1 INTRODUCTION

Understanding the interplay between demographic and environmental processes that shape mating systems has long been a major focus of evolutionary ecology (Kokko, Klug, & Jennions, 2012; Kokko & Rankin, 2006; Manning, Medill, & McLoughlin, 2015; Simmons & Kvarnemo, 2006; Székely, Weissing, & Komdeur, 2014). In particular, the social and ecological environments have been presumed to influence the potential of males to monopolize mates (Emlen & Oring, 1977). Accordingly, Emlen and Oring (1977) proposed that the sex ratio was a reproductive competition coefficient among mating males, such that a greater number of breeding males relative to receptive females led to an increased intensity in male competition for mates. These authors also introduced the Environmental Potential for Polygyny (EPP) hypothesis, which predicts that resource availability represents a major determinant of mating inequality (Emlen & Oring, 1977). The EPP hypothesis postulates that females generally tend toward a clustered distribution around spatially clustered resources, allowing males to defend a resource patch and monopolize multiple females (i.e., resource defense polygyny). It further predicts that the degree of male mating success decreases when females are sparsely dispersed across uniform environments. The EPP perspective established the foundation for many of our current views of animal mating systems (see Shuster & Wade, 2003).

Despite the EPP hypothesis being widely accepted, complex spatiotemporal and scale‐dependent processes operate within populations (Kokko & Rankin, 2006), making it difficult to quantify the EPP (Shuster, 2009). For example, emerging evidence suggests that mate choice can be highly variable and dependent upon the prevailing weather conditions (Robinson, Doorn, Gustafsson, & Qvarnström, 2012). Weather has also been shown to interact with resource availability along environmental gradients to drive spatially dynamic adult sex ratios (ASRs) and polygyny thresholds (Manning & McLoughlin, 2017; Manning et al., 2015). Additionally, variation in population density may have sex‐specific effects (see Kokko & Rankin, 2006), and density‐dependent processes have been shown to be strongly scale‐dependent (Ray & Hastings, 1996). Considering such complexities, Kokko and Rankin (2006) highlighted the importance of considering the joint effects of mating systems and population demography in studies of sexual selection, and Shuster (2009) developed an approach to quantify and account for the spatial and temporal distribution of mating within populations. Subsequently, the opportunity for sexual selection was shown to vary among sequential reproductive episodes (e.g., the selection of breeding sites and subsequent acquisition of mates) further demonstrating the underlying complexities of sexual selection (Klug, Lindström, & Kokko, 2010). The recognition that demographic and environmental processes may operate in concert with ASR to shape varying degrees of opportunity for sexual selection forms an important basis from which to test competing hypotheses regarding the drivers of male mating success across sequential reproductive episodes within natural populations.

Building on the framework of Emlen and Oring (1977) and Shuster (2009), we investigated the differential effects of spatiotemporally varying environmental and demographic factors on male mating success across selection episodes in a naturalized population of feral horses (*Equus ferus caballus*) in Sable Island National Park Preserve, Canada. Horses were introduced to Sable Island in the mid‐1700s and have remained feral throughout their history. Male feral horses compete to attain and retain females, and the degree of polygyny varies across environmental gradients characterized by the distribution of limiting resources (e.g., freshwater; Manning & McLoughlin, 2017).

We tested competing hypotheses regarding resource availability, weather, and spatiotemporal demographic variability on male mating success. We also accounted for ASR and conducted this study across multiple selection episodes using a spatial and longitudinal 5‐year mark‐resight dataset. We expected resource availability effects on male mating inequality because male horses do not possess ornaments and there is little or no sexual dimorphism (Berger, 1986), indicating the importance of resource quality in female mate choice and the presence of the EPP. We expected density effects on male mating inequality in both selection episodes, as density has been linked to ASRs in this system (Manning et al., 2015). We predicted that limited freshwater availability would increase male mating inequality in the second episode because increased water requirements during reproduction, pregnancy, and lactation would heighten female mate choice under these conditions (Contasti, Tissier, Johnstone, & McLoughlin, 2012; Richard, Simpson, Medill, & McLoughlin, 2014). Lastly, in line with the EPP hypothesis, we predicted that a freshwater availability effect on male mating inequality would vary among years, as the annual amount of rainfall that recharges the island's water table and influences freshwater availability has been shown to underlie spatially dynamic polygyny thresholds in this system (Manning & McLoughlin, 2017).

## 2 METHODS

### 2.1 Study area and system

**Figure 1:**
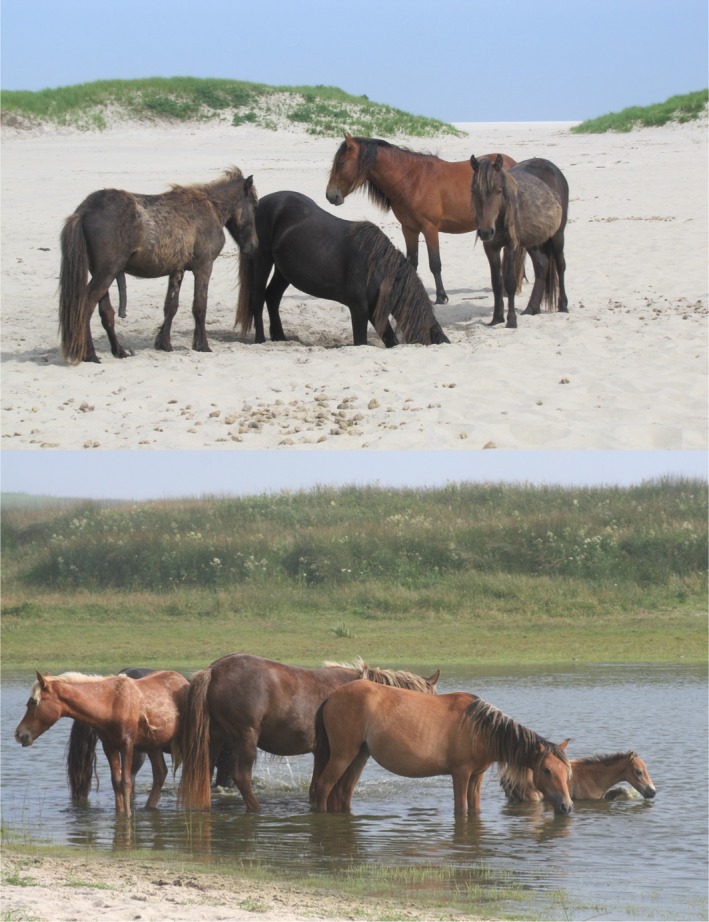
A gradient in freshwater availability leads to feral horses that dig and wait for water on the xeric side (top) and frolic and share water on the mesic side (bottom) of Sable Island, Canada. Photos ©Jeffrey A. Manning

Sable Island National Park Preserve is an emergent 49‐km long by 1.5‐km wide sandbar located 275 km southeast of Halifax, Nova Scotia, Canada (43°55′N, 60°00′W). East–west resource gradients (i.e., in availability of freshwater and forage; Figure 1) across the island's length underlie spatially heterogeneous demographic rates and density‐dependent habitat selection processes in this horse population (Contasti, van Beest, Vander Wal, & McLoughlin, 2013). The horses have been completely isolated from introgression since 1935 and are without confounding effects of predation, human interference, or interspecific competition (they are unmanaged and the island's only terrestrial mammal; Christie, 1995; Plante et al., 2007). Like other species (e.g., lance‐tailed manakin—McDonald, 2007; DuVal & Kemenaers, 2008), the opportunity for sexual selection on male horses follows two sequential episodes, which we considered in this study: (a) male–male competition among all males of breeding status to acquire or hold a harem; and (b) male–male competition among only dominant males coupled with female mate choice to acquire additional females once a harem has been acquired. These conditions have major consequences for the island's horse population and social dynamics (van Beest et al., 2014), making this system highly suitable for investigating aspects of the EPP hypothesis across selection episodes.

Feral horses form stable, year‐round social and breeding groups (bands) consisting of a dominant male (stallion) with a harem of females, similar to that of some primates, and are tenacious to undefended home ranges that overlap those of other bands (Berger, 1986; Linklater, Cameron, Minot, & Stafford, 1999). Bands make regular forays between foraging and watering areas (Berger, 1986; Manning et al., 2015), producing a male mating system governed by both resource and female defense polygyny (Emlen & Oring, 1977; Manning et al., 2015).

Maturing males may remain in the natal band, move to another band, or join groups of unpaired males that range widely (Asa, 1999; Contasti et al., 2012). Males begin to acquire and defend harems at ≥4 years of age (Berger, 1986; Contasti et al., 2012; Hoffmann, 1985; Welsh, 1975). Some males that are old enough to mate do not hold any defined social status (bachelors), while few enter into long‐term social alliances with dominant band stallions. These subordinate partners (tags) have been found to mate with females within their harem, although rarely (Berger, 1986; Linklater et al., 1999; Welsh, 1975). Consequently, only a proportion of the male population monopolizes mating, which constitutes one of the few cases where sex ratio and the opportunity for sexual selection may accurately predict sexual selection (Klug, Heuschele, Jennions, & Kokko, 2010).

Harem tending by dominant males occurs throughout much of the year, with agonistic interactions and defecation‐marking occurring year‐round (Asa, 1999; Berger, 1986; Hoffmann, 1985; Welsh, 1975). Harems are relatively stable during the breeding season, although the composition of harems changes annually due to breeding dispersal at rates of 0.16 for adults and 0.34 per year for 1‐ to 3‐year‐olds (Marjamäki, Contasti, Coulson, & McLoughlin, 2013). Unpaired males may also influence selection of males via increased male–male aggression events, dominant male harassment toward mares, and potential yet rare sneak matings (Berger, 1986; Linklater et al., 1999; Smuts & Smuts, 1993). A more detailed description of the Sable Island population can be found elsewhere (van Beest et al., 2014; Contasti et al., 2013, 2012; Marjamäki et al., 2013).

### 2.2 Data collection

**Figure 2:**
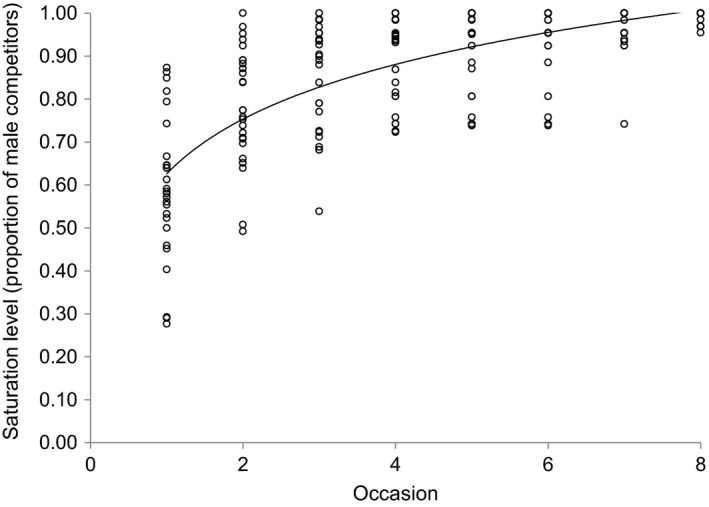
The saturation curve of the proportion of competitor males (dominant band stallions and unpaired bachelors ≥ 4 years old) encountered by dominant male wild horses (*Equus ferus caballus*) across consecutive sampling occasions within a breeding season. The equation (*y* = 0.184ln(*x*) + 0.626; *r* ^2^ = 0.565) estimates that dominant band males encounter all competitor males by the 8th occasion, indicating adequate sampling effort. Black circles represent dominant males. Data are from 27 dominant males tending harems on Sable Island, Canada, 2008–2012

From 2008 to 2012, we collected individual‐based field observations (*n* = 10,921) of the entire horse population during systematic, daily ground surveys each spring breeding season (*n* = 5 Table 1). Individuals were identified using digital photographs. Each record consisted of the date of observation, geographic coordinates, sex, age, reproductive status, and group affiliation. High‐resolution aerial photography from January 2010 was used to confirm that population surveys accounted for >99% of the horses found on the island (Richard et al., 2014). Although feral, horses were neither fearful of humans nor accustomed to rewards, allowing us to approach each individual and record data without disturbing or altering behaviors. All individuals affiliated with a group were assigned the same location. Each breeding season survey consisted of a series of 8‐ to 10‐day island‐wide censuses, which effectively accounted for the number of male competitors that dominant males encountered (Figure 2). We classified age as foals (age 0), yearlings (1‐year old), young adults (2–3 years), or mature horses (≥4 years) based on known ages of birth for animals born on or after 2007 (Contasti et al., 2012). We included young adults in our reference to “adult” or “breeding” females because females bred only at age ≥2 (giving birth for the first time at age 3). However, we include only males aged ≥4 years in our definition of “adult” males (both dominant males and unpaired males), as sexual activity in male feral horses aged ≤4 years is rarely observed (Hoffmann, 1985).

**Table 1:** Annual number of wild horse individuals, adult males, adult females, harems, and harem size (females per harem) on Sable Island, Canada, 2008–2012 (see “Materials and methods”)

Year^a^	Population^b^	Adults		Harems	Harem size ($\bar{x}$ ± 95% CI)
		Males	Females		
2008	375	137	134	52	2.60 ± 0.59
2009	425	152	143	58	2.71 ± 0.42
2010	484	167	165	73	2.33 ± 0.35
2011	450	183	145	73	2.12 ± 0.27
2012	534	214	175	79	2.32 ± 0.34

a Data from 2008 to 2010 gathered by Contasti et al. (2012).

b Number of individuals of all age classes as computed by van Beest et al. (2014).

### 2.3 Estimates of mating success

Mating success for each dominant male was defined as the number of females qualified to mate (≥2 years) within a band during each breeding season (Berger, 1986; Lucas, Raeside, & Betteridge, 1991; Shuster & Wade, 2003). We considered adult female abundance as a qualified index of mating success because pregnancy rates are typically high in this population (>75%; Welsh, 1975), leading to high rates of population growth (Contasti et al., 2013) and a correlation between the number of pregnancies and females. We assigned mating success only to dominant males, including those in bands where a tag male was present (<0.05% of our observations). Here, all animals termed “dominant” possessed a harem (we use the term to reflect “alpha” status within a band).

To investigate mating inequality among males within the population, we calculated georeferenced estimates of standardized variance in male mating success (*I* = variance/[mean mating success^2^]) in each band during each breeding season (Arnold & Wade, 1984; Shuster & Wade, 2003). As calculated, the *I* is an empirical measure of variance in relative mating success, which does not require precise knowledge of the mechanism by which selection occurs or the specific traits that are associated with fitness variance (Arnold & Wade, 1984; Crow, 1958; Hersch & Phillips, 2004; Shuster & Wade, 2003). It builds on the conceptual framework of Emlen and Oring (1977), effectively enabling us to examine the spatiotemporal distribution of opportunity for sexual selection within the population (Arnold & Duvall, 1994; Crow, 1958; Emlen & Oring, 1977; Klug, Heuschele et al., 2010; Krakauer, Webster, Duval, Jones, & Shuster, 2011; Shuster, 2009).

We carried out the following steps to calculate band‐specific estimates of *I*. First, we used the median maximum distance moved by horses during the breeding season (2.06 km; Manning et al., 2015) to buffer the locations of all bands. This small buffer size was supported by the fine‐scale genetic substructure found in this population across the island (Lucas, McLoughlin, Coltman, & Barber, 2009). Second, we counted all dominant males (including subordinate tag males) and females qualified to mate that had overlapping locations with a band's set of buffered locations. Third, we used these counts of males and females to calculate a single, spatially explicit *I* for each band during each breeding season.

We extend the variance of *I* (the squared number of mates per male minus the average number of mates per male squared; Sokal & Rohlf, 1995) to account for our spatiotemporal data, such that.

(1) $${\text{variance}}_{m_{k}}=\sum_{k=1}^{n}\left(f_{\mathrm{ij}}^{2}m_{\mathrm{ij}}\right)/\sum_{k=1}^{n}m_{\mathrm{ij}}-{\left[\sum_{k=1}^{n}\left(f_{\mathrm{ij}}m_{\mathrm{ij}}\right)/\sum_{k=1}^{n}m_{\mathrm{ij}}\right]}^{2}$$

and

(2) $$\text{mean}\;\text{mating}\;{\text{success}}_{k}=\sum_{k=1}^{n}\left(f_{\mathrm{ij}}m_{\mathrm{ij}}\right)/\sum_{k=1}^{n}m_{\mathrm{ij}},$$

where *m_ij_* is the number of dominant males having overlapping space use (i.e., present within ≥1 locations) with dominant male *i* in breeding season *j*, and *f_ij_* denotes the number of females mated (i.e., harem size) with dominant male *i* in breeding season *j *for *k* = 1, …, *n* bands. By calculating *I* for individual dominant males, each of which generally does not overlap or co‐occurs with the same set of dominant males and harems, we effectively estimate the variance in mating success at local sites (e.g., population segments) across the island.

As calculated, *I* is a dimensionless value that reflects the theoretical maximum potential strength of sexual selection at a site (Klug, Lindström et al., 2010; Krakauer et al., 2011). Estimates of *I* also capture stochastic variation. Despite the view that stochastic variation is undesirable in estimates of opportunity for sexual selection (Jennions, Kokko, & Klug, 2012; Klug, Lindström et al., 2010), stochastic events that influence the trajectory of demographic rates are often linked to environmental and/or demographic variation that is measureable and likely deterministic (see Festa‐Bianchet, Coulson, Gaillard, Hogg, & Pelletier, 2006; Frederiksen, Daunt, Harris, & Wanless, 2008), making this metric well suited for disentangling demographic and environmental effects on mating inequality.

For each dominant male, we further partitioned *I* into its sequential, competitive episodes separately during each breeding season (Ahnesjö, Kvarnemo, & Merilaita, 2001). The opportunity for sexual selection associated with acquiring a harem (*I*_harem_) was calculated as the variance in success that males had in acquiring a harem (0 or 1) divided by the square of the mean success of harem acquisition. All qualified males (dominant males, tags, and bachelors that were old enough to mate (≥4 years; Berger, 1986; Lucas et al., 1991)) in a buffered location had the opportunity to compete for harems (Berger, 1986; Linklater et al., 1999; Smuts & Smuts, 1993), and were included in these calculations. The opportunity for selection associated with acquiring mates once a male attained a harem (*I*_mares|harem_) was computed as the variance in mating success, that is, the number of qualified mares each male had in his harem (which equals the number of qualified mares in a band) divided by the mean mating success. Only dominant males acquire mates and were included in *I*_mares|harem_.

### 2.4 Statistical analysis

#### 2.4.1 Island‐wide variability in mating success

We began by testing for annual patterns in island‐wide abundance of harems and population size using a general linear model (LM). To test for differences in male mating success among years, we used the Brown‐Forsythe Levene‐type test of equality of variances in the lawstat package (Noguchi, Hui, Gel, Gastwirth, & Miao, 2013) in R (R Development Core Team, 2012). We performed this test on *I* and both selective episodes separately. We also tested whether the island‐wide mean mating success differed among years separately for *I* and each selective episode using Kruskal–Wallis rank sum tests (Hollander, Wolfe, & Chicken, 2014), with individual adult males as the sampling unit.

#### 2.4.2 Models of ASR on mating success

We modeled variation in mating success among dominant males attributed directly to local ASR for each selection episode using hierarchical mixed‐effects linear models (LMM). For each dominant male, we estimated local ASR (ASR*_ij_*) as the mean number of adult males/(adult males + adult females) with ≥1 buffered locations that overlapped ≥1 locations of the *i*th band over the course of the field season in the *j*th year (Manning et al., 2015; Parker & Simmons, 1996). This relative ASR was spatially and temporally linked with its corresponding estimate of *I*, included unpaired adult males, and ranged from 0 when only females were ready to mate, through 0.5 at equality, to 1 when only males were ready to mate.

As demonstrated with our other work in this system (see Manning et al., 2015; Manning & McLoughlin, 2017), some horses were observed repeatedly over the study while others were observed only once in a season, leading to samples that were unequally distributed among the dominant males. Repeated counts of individuals within each year $\left(\bar{x}=2.84,SD=1.43\right)$ were accounted for by first using the sum number of unique overlapping individuals in a given year to calculate annual measures of each selection metric associated with a dominant male, and also including individual male identity as the random effect intercept term in each model (Laird & Ware, 1982). This design allowed analyses to include a measure of variability among dominant males as well as among years.

Using ASR*_ij_* as an explanatory variable, we constructed a separate LMM for each selective episode, with *I*, *I*_harem_, and *I*_mares|harem_ treated as a response. We used the lme4 package in R version 2.14.1 (R Development Core Team, 2012) to calculate descriptive statistics and obtain mixed‐effects model estimates using maximum likelihood (Harville, 1977). These LMMs, following the notation of Gelman and Hill (2007), took the form of $y=\alpha_{kj[i]}+\beta_{0}+\beta_{x-x[i]}+\varepsilon_{kj[i]}$, where *y* represents the dependent variable, the random effect intercept of $\propto_{kj[i]}∼N\left(\mu =0,\sigma_{k}^{2}\right)$ for the *k*th dominant male in year *j* for *i* = 1, …, n observations, $\beta_{0}$ is the fixed effect intercept, $\beta_{x}$ is the rate of fixed effect *x*, and ε is the error. We used the MuMIn package in R version 3.0.2 (R Development Core Team, 2012) to calculate the marginal *R* ^2^ for each model, which described only the proportion of variance explained by the fixed factor(s) (Johnson, 2014; Nakagawa & Schielzeth, 2013), and the lmerTest package to calculate degrees of freedom, *F*‐statistics, and *P*‐values based on the Satterthwaite's approximations (Goodnight, 1976). We also used the HLMdiag package in R to extract the standardized marginal residuals from each model for use in the following sets of models.

#### 2.4.3 Models of environment and demography on mating success

We constructed a second candidate set of models to test competing hypotheses regarding environmental and demographic factors (resources, total density, and density of unpaired adult males) that may affect male mating success during each selection episode. This second set of LMMs (residual‐based LMMs) followed the same form as above, but with the marginal residuals that we extracted from the previous corresponding ASR‐LMMs as the response for *I* and each episode (*I*_harem_, and *I*_mares|harem_) separately (see Jakob, Marshall, & Uetz, 1996). As such, these marginal residuals represented the variation in mating success that remained after accounting for effects of ASR*_ij_*.

A preliminary analysis of marginal residuals against fitted values and normal QQ plots indicated that residuals were satisfactory for such an analysis (i.e., they were not intercorrelated or heteroscedastic; Singer, Nobre, & Rocha, 2013). Although the use of residuals in comparing among groups under particular circumstances has been challenged (García‐Berthou, 2001), our approach applied residuals as a continuous unbounded response in mixed regression models, effectively considering marginal residuals as the variation remaining beyond that explained by ASR alone. Their inclusion as the response variable enabled us to effectively assess the direct effects of environmental and demographic factors anticipated to influence mating success after controlling for ASR and random effects.

To test our prediction that freshwater availability effects would differ among years due to annual variation in rainfall, we included a year effect in all residual‐based LMMs because we previously found interannual effects of summer precipitation on the availability of ephemeral water sources and spatially explicit ASRs in this system (Manning et al., 2015). Following Manning et al. (2015), we calculated a yearly summer precipitation index (i.e., 2008 = −0.747, 2009 = 0.878, 2010 = −1.239, 2011 = 0.339, and 2012 = −0.545). We compared the relative fit of the year‐effects model to a null and used year‐effects as the baseline structure from which to further develop 2‐predictor‐variable models comprised of continuous variables anticipated to shape the spatial and temporal variation in male mating success (Table 2). We also included distance to water as a predictor in some residual‐based LMMs. Here, we used the mean Euclidean distance (km) from the nearest permanent freshwater pond to within‐summer locations of each band (acquired from an existing digital dataset of freshwater pond locations; Contasti et al., 2012). If year and distance from water improved model fit, we further tested for effects of summer precipitation on the rate at which annual mating inequality changed with distance from water. For this, we used the rate at which the opportunity for selection changed with distance from water (obtained as the year‐specific coefficients from the associated LMM) as a polynomial (concave or convex) function of summer precipitation, and predicted extreme rates during years characterized by extreme summer precipitation.

**Table 2:** Effects of spatially heterogeneous resources, total density, and density of unpaired adult males (after accounting for ASR) on specific episodes of opportunity for male selection (total *I*, *I*_harem_, and *I*_mares|harem_) for 335 repeat observations of 118 wild harem‐holding male horses on Sable Island, Canada, 2008–2012. Estimates are shown from hierarchical linear mixed‐effects regression considering individual male identity as a random effect and accounting for potential effects of adult sex ratio and annual variation

Models	*k* a	ΔAICc^b^	*w_i_* c	Log‐likelihood	*R* c
Linear mixed‐effects models of total male selection (*I*)					
Distance from permanent freshwater × year	12	0	1.00	343.9	0.45
Unpaired adult male density × year	12	37.29	0.00	325.2	0.41
Total density × year	12	47.76	0.00	320.0	0.39
Year	7	117.49	0.00	279.8	0.21
Null	3	194.59	0.00	237.1	
Linear mixed‐effects models of male selection associated with acquiring a harem (*I*_harem_)					
Unpaired adult male density × year	12	0	1.00	604.0	0.57
Total density × year	12	66.63	0.00	570.7	0.49
Distance from permanent freshwater × year	12	102.58	0.00	552.7	0.38
Year	7	147.20	0.00	525.1	0.29
Null	3	270.62	0.00	459.3	
Linear mixed‐effects models of male selection associated with acquiring mates (*I*_mares|harem_)					
Distance from permanent freshwater × year	12	0	1.00	306.7	0.41
Unpaired adult male density × year	12	51.57	0.00	281.0	0.33
Total density × year	12	58.91	0.00	277.3	0.31
Year	7	114.33	0.00	244.3	0.15
Null	3	168.38	0.00	213.1	

a Degrees of freedom.

b Second‐order Akaike's information criterion.

c AICc weight.

Marginal *R* ^2^: describes only the proportion of variance explained by the fixed factor(s) (Johnson, 2014; Nakagawa & Schielzeth, 2013).

Density of unpaired adult males was measured as the total number of unpaired adult males over an entire breeding season per km^2^ of terrestrial area across a band's buffered locations, and total density was calculated as the cumulative count of unique horses (all ages, sexes, and social classes) over an entire breeding season per km^2^ of terrestrial area across a band's buffered locations. Observations of individual bands were not averaged over the entire 5‐year period because potentially important variables of interest relating to resources and local density often varied annually.

Within each set of residual‐based LMMs associated with a given selection episode, we used second‐order Akaike's information criterion (AICc) weights (*w*_AICc_) to quantify the support of each model, where *w*_AICc_ can be interpreted as the probability that a specific model is the best in the candidate set (Burnham & Anderson, 2002). We also used the marginal *R* ^2^ (Johnson, 2014; Nakagawa & Schielzeth, 2013) to assess the direct contribution of the general effects in each model on *I* and its subcomponents *I*_harem_ and *I*_mares|harem_. Additionally, for each episode, we tested whether our final residual‐based LMM accounted for spatial autocorrelation structure among males that may emerge as a result of nonindependence. We did this because individual‐based approaches like this may introduce nonindependence in the counts of male competitors whose spatial locations overlap. For this, we controlled for location and tested if the inclusion of a Gaussian correlation structure in our final residual‐based LMMs improved model fit. Statistical analyses were computed in R (R Development Core Team, 2012) and ArcGIS (version 10.0).

## 3 RESULTS

### 3.1 Island‐wide variability

Mean, island‐wide total opportunity for male selection differed among years (Kruskal–Wallis: *K*_4_ = 111.1, *p* < 0.001), although there was little evidence of yearly differences among males across the island (Brown‐Forsythe Levene: *W* = 2.3, *p* = 0.06). Island‐wide, *I*_harem_ *,* differed spatially and across years (Brown‐Forsythe Levene: *W* = 13.9, *p* < 0.001; Kruskal–Wallis: *K*_4_ = 183.4, *p* < 0.001). There was also a difference among years in mean *I*_mares|harem_ (Kruskal–Wallis: *K*_4_ = 102.9, *p* < 0.001), but no annual difference among males in this metric (Brown‐Forsythe Levene: *W* = 0.76, *p* = 0.55). Abundance of harems on the island increased annually with total population size (lm: *R* ^2^ = 0.87, *F*_1,3 = _20.7, *p* = 0.02; Table 1).

### 3.2 ASR effects

**Figure 3:**
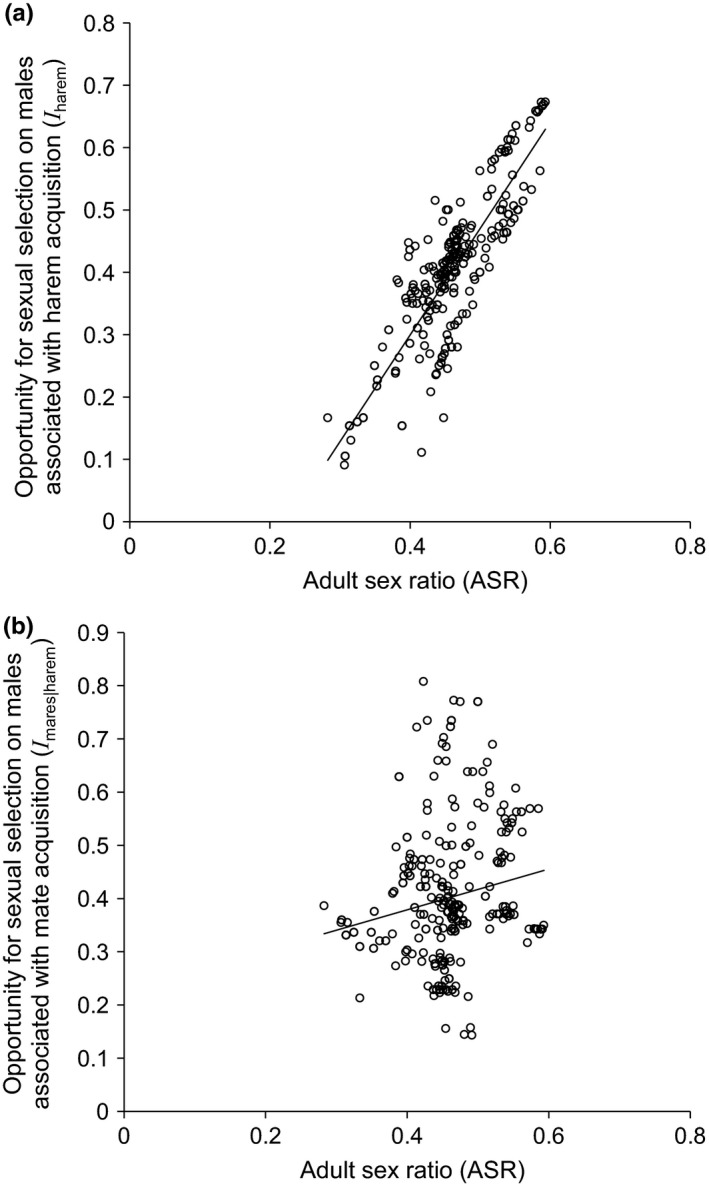
Adult sex ratio in relation to opportunity for sexual selection on male wild horses (*Equus ferus caballus*) associated with (a) acquiring a harem (lmm: *y* = 1.725*x* − 0.392; *F*_1, 310_ = 994.2, *p* < 0.001, *R* ^2^ = 0.74) and (b) mate acquisition (lmm: *y* = 0.396*x* + 0.220; *F*_1, 310_ = 11.93, *p* = 0.001, *R* ^2^ = 0.03). Data are from locations (*N* = 335) of individual males (*N* = 118) on Sable Island, Canada, 2008–2012

Adult sex ratios explained a significant but small amount of total opportunity for sexual selection on males (lmm: *I*: *F*_1, 310_ = 17.21, *p* < 0.001, marginal *R* ^2^ = 0.05). Specifically, a 10% increase in ASR increased total opportunity for selection by 44% (*t* = 4.15, *p* < 0.001). Of the two selective episodes considered, ASR had the strongest effect on harem acquisition (lmm: *I*_harem_: *y* = 1.725*x* − 0.392; *F*_1, 310_ = 994.2, *p* < 0.001; Figure 3a), explaining 74% of the variation. Although ASR influenced the opportunity for male selection during mate acquisition (lmm: *I*_mares|harem_: *y* = 0.396*x* + 0.220; *F*_1, 310_ = 11.93, *p* = 0.001; Figure 3b), it explained only a small portion of that variation (marginal *R* ^2^ = 0.03).

### 3.3 Environmental and demographic effects

Consistent with our predictions, resources and densities differed in their effects on *I*, *I*_harem_, and *I*_mares|harem_ among selection episodes, and there was little evidence that controlling for a correlation structure associated with nonindependence among male competitors improved the fit of the best models in each episode (ΔAICc for the model with a Gaussian correlation structure in each episode was >2.0 from the associated best fit model). There was strong evidence that *I* varied among years (ΔAICc between the Year_AICc_ and Null_AICc_ models = 77.10; Table 2). After accounting for ASR, yearly differences, and random effects, distance from permanent freshwater was the most important determinant of the remaining variation in total opportunity for selection on males (lmm: *I*—predicted by distance from permanent freshwater × year: *w*_AICc_ = 1.0, marginal *R* ^2^ = 0.16; Table 2).

**Figure 4:**
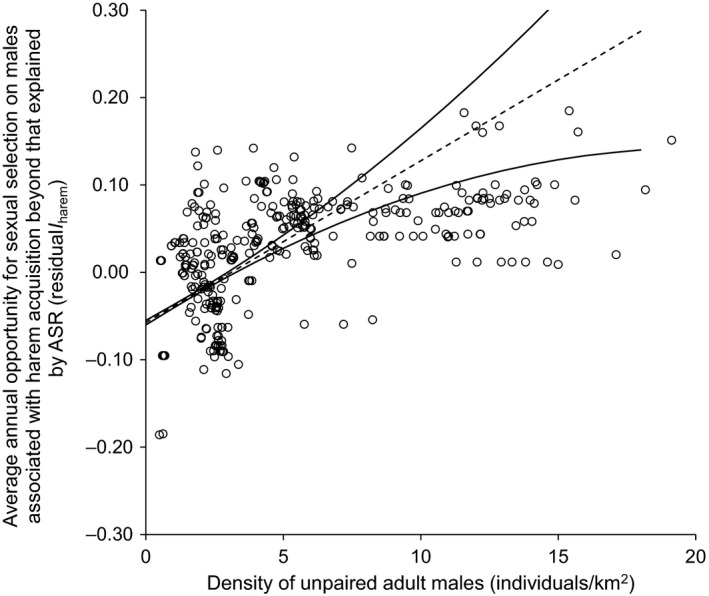
Predicted average annual variability in opportunity for sexual selection associated with harem acquisition (dotted line) on male wild horses (*Equus ferus caballus*) beyond that explained by adult sex ratio (ASR) in relation to density of unpaired adult males, with interannual variance (solid lines). Predictions are shown from supported mixed‐effects linear regression considering individual male identity as a random effect. Circles are marginal residuals of the opportunity of sexual selection after accounting for ASR in prior mixed‐effects linear model; the larger the absolute value of the residual, the farther from expected opportunity of selection attributed to ASR a male is. Data are from locations (*N* = 335) of individual males (*N* = 118) on Sable Island, Canada, 2008–2012

After accounting for ASR, yearly variation in *I*_harem_ and *I*_mares|harem_ was evident, with differing factors emerging as important predictors in each episode (Table 2). After accounting for ASR, yearly differences, and random effects, there was still substantial opportunity for male selection remaining in *I*_harem_, with strong evidence that density of unpaired adult males explained 32% of that remaining variation (lmm: *I*_harem_ bachelors: *w*_AICc_ = 1.0, marginal *R* ^2^ = 0.32; Table 2). There was no evidence that *I*_harem_ varied according to total density or distance from water (*w*_AICc_ = 0.0). Increasing density of unpaired adult males increased the residual variation in *I*_harem_ in all years. Specifically, an average increase in one unpaired adult male per km^2^ increased residual variation in *I*_harem_ by 10%, indicating an increased importance of unpaired male abundance on *I*_harem_ (Figure 4). The preponderance of males occurring in locations supporting a high density of unpaired males provided greater opportunity for male selection pressure during harem acquisition than expected by ASR (i.e., positive residuals; Figure 4).

**Figure 5:**
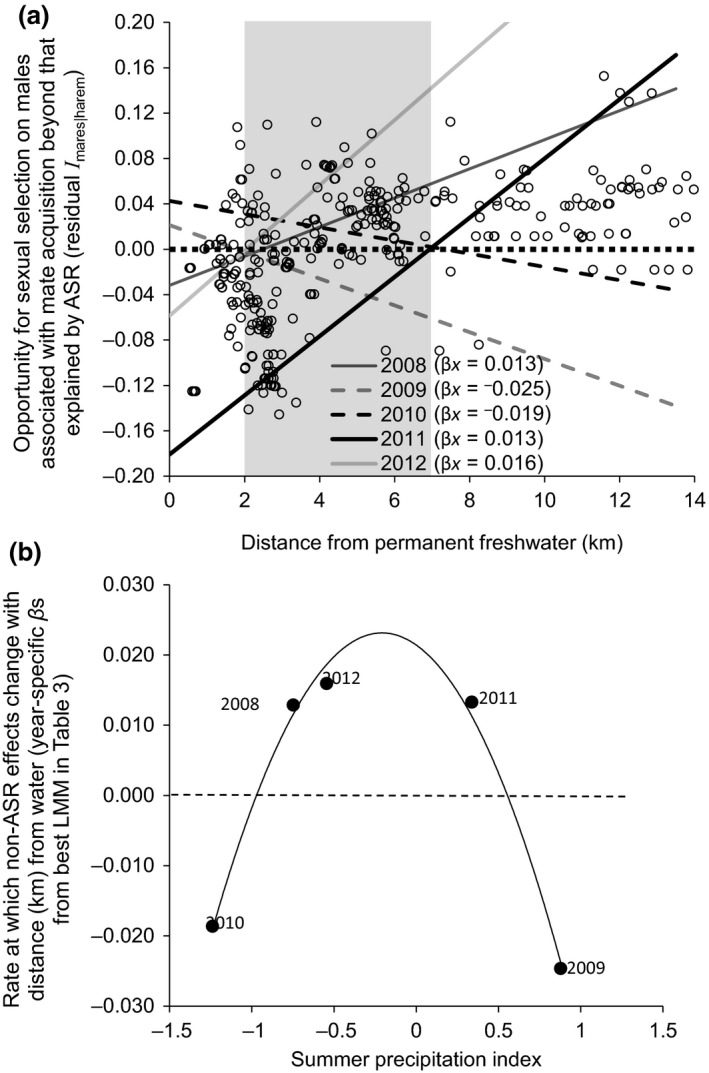
Predicted (a) opportunity for sexual selection on male wild horses (*Equus ferus caballus*) associated with mate acquisition beyond that accounted for by adult sex ratio (ASR) in relation to availability of a limiting resource (i.e., distance from permanent freshwater), and (b) rate at which these non‐ASR effects that change with distance (km) from freshwater (*β*; obtained from post hoc analysis as the year‐specific coefficients from the associated LMM in Table 3 and Figure 5a) vary as a polynomial function of summer precipitation (*y* = 0.021 − 0.017*x *− 0.040*x* ^2^, *F*_2, 2_ = 117.7, *p* = 0.008, *R* ^2^ = 0.98). Predictions are shown from supported mixed‐effects linear regression considering individual male identity as a random effect. In (a), circles are marginal residuals of the opportunity of sexual selection after accounting for ASR in a prior mixed‐effects linear model, ASR explains much of the opportunity for selection when *residual I*_mares|harem_ = 0, less than expected when the residual >0, and more than expected when the residual <0, with *residual I*_mares|harem_ becoming exceedingly biased on average at distances <2 and >7 km (shaded area) from permanent freshwater. Data are from locations (*N* = 335) of individual males (*N* = 118) on Sable Island, Canada, 2008–2012 (see “Materials and methods”).

In contrast, distance from permanent water was the strongest determinant of residual variation in *I*_mares|harem_ (lmm: *I*_mares|harem_ permanent water: *w*_AICc_ = 1.0, *R* ^2^ = 0.17; Figure 5a), with no evidence that *I*_mares|harem_ varied according to bachelor density or total density (Table 2). Additionally, the rate and direction of this relationship depended on the amount of summer precipitation (Figure 5a). Specifically, during years with average summer precipitation (summer precipitation index < |0.8|; 2008, 2011, 2012), the variation in *I*_mares|harem_ that was unaccounted for by ASR increased positively at increasing distance from permanent water, indicating that *I*_mares|harem_ was greater than predicted by ASR at sites farther from water (i.e., ASR alone predicted biased low estimates of *I*_mares|harem_ far from water; Figure 5a). The opposite pattern emerged during years with extreme summer precipitation levels (summer precipitation index > |0.8|; 2009, 2010), such that sites farther from permanent water had less opportunity for male selection associated with acquiring mates than expected by ASR (i.e., ASR led to biased high estimates of *I*_mares|harem_ far from water; Figure 5a).

For both average and extreme summer precipitation years, the residual variation in *I*_mares|harem_ was predicted to reach zero (i.e., where ASR was an unbiased estimator of *I*_mares|harem_) at sites between 2 and 7 km from permanent water, and estimates of *I*_mares|harem_ determined by ASR became exceedingly biased at distances <2 and >7 km from permanent freshwater in our system (Figure 5a). Summer precipitation also appeared to influence whether this bias was positive or negative along the gradient in distances from water, as ASR overestimated *I*_mares|harem_ at sites <2 km from water and underestimated *I*_mares|harem_ at sites >7 km during years with average summer precipitation, whereas the opposite pattern emerged during extreme precipitation years (Figure 5a). A post hoc analysis revealed that total summer precipitation was the best predictor of *I*_mares|harem_ compared to other precipitation and temperature variables (see Table 3 for details). Moreover, this analysis showed that summer precipitation further influenced the annual rate at which *I*_mares|harem_ changed with distance from water, following a concave pattern with extreme negative rates during years characterized by extreme precipitation levels (*R^2^* = 0.98; Table 3, Figure 5b).

**Table 3:** Results from post hoc analysis. Effects of climate on the annual rate at which male selection associated with acquiring mates (*I*_mares|harem_) changes with distance (km) from freshwater for 335 repeated observations of 118 wild harem‐holding male horses on Sable Island, Canada, 2008–2012. Annual rates are from linear mixed models of distance to water on residual effects beyond that explained by ASR (see Figure 5a). Climate variables included in models were based on previous work on horses in this system (e.g., Contasti et al., 2012; Manning et al., 2015; Manning & McLoughlin, 2017). Estimates are shown from linear polynomial regression models

Models	*k* a	ΔAICc^b^	*w_i_* c	Log‐likelihood	*R* ^2^
Total summer precipitation + total summer precipitation^2^	3	0	0.97	18.3	0.98
Mean summer maximum temp + mean summer maximum temp^2^	3	7.73	0.02	17.1	0.92
Total winter precipitation + total winter precipitation^2^	3	18.40	<0.01	15.7	0.33
Total winter precipitation	2	18.53	<0.01	14.7	0.31
Mean winter minimum temp	2	21.50	<0.01	13.2	0.23
Total summer precipitation	2	21.70	<0.01	13.1	0.23
Mean summer maximum temp	2	21.82	<0.01	13.1	0.23
Mean winter minimum temp + mean winter minimum temp^2^	3	22.49	<0.01	12.0	0.24

a Degrees of freedom.

b Second‐order Akaike's information criterion.

c AICc weight.

## 4 DISCUSSION

Using spatially and temporally heterogeneous demographic and environmental data, we found a disparity in the effect of ASR on opportunities for sexual selection among sequential selective episodes. Although ASR did contribute to the opportunity for male selection associated with acquiring a harem, this finding may be attributed to both ASR and the opportunity for male selection associated with acquiring a harem being expressed as a function of male–male competition among paired and unpaired males. This may have occurred because the presence of unpaired males may strongly affect the mating decisions in a population even if these individuals do not mate or care for young (Webb, Székely, Houston, & McNamara, 2002). In contrast, ASR had little effect on total opportunity for selection and that associated with acquiring females after securing a harem. The spatial dynamics of freshwater availability, an important resource for females, appears to play an important role in female mate choice and the ability of a male to acquire mates, effectively swamping out the influence of ASR on male mating inequality. Regardless of mechanism, our results confirm the notion that sex ratio is not a consistent predictor of opportunity for selection, nor does it operate alone in determining male mating success (Székely et al., 2014).

We also found flexibility in the magnitude of opportunity for selection in males among selective episodes, a finding consistent with Klug, Lindström et al. (2010). Although variation in total opportunity for selection across the population (i.e., the island) remains constant among years, we show that the mean and variability in magnitude of opportunity for selection can differ with respect to the selective episode considered. Specifically, the mean and within‐year variation in *I*_harem_ across the island depended on the year. In contrast, once a harem was acquired, dominant males encountered a different mean opportunity for acquiring mares each year. These annual patterns appear to be related to weather (see Twiss, Thomas, Poland, Graves, & Pomeroy, 2007) and suggest the importance of demographic and environmental hypotheses in which annual meteorological conditions can interact with a spatially dispersed limiting resource and unpaired male densities to create variable magnitudes of opportunity for selection among males. One particular aspect of demography that could possibly bias our estimates of *I*_mares|harem_ low is the presence of tags and bachelors of breeding age that mated with a female, although tags and such mating events are rare (tags present in <0.05% of our observations, and also rare in other systems; Welsh, 1975; Berger, 1986; Linklater et al., 1999). Additionally, such rare events likely have little effect on our results that show varying degrees of importance and bias of ASR on *I*_mares|harem_ attributed to the interaction between meteorological conditions and the gradient in freshwater availability. This finding raises interesting theoretical and conservation‐related questions for this population in light of projected increases in the interannual variability of precipitation and temperature in the North Atlantic region.

The differential effects of ASR, unpaired male abundance, freshwater availability, and summer precipitation we found on the opportunity for male selection across selection episodes on Sable Island demonstrate an increased complexity in our former understanding of the EPP model. For instance, distance from permanent water and summer precipitation jointly contributed to *I*_mares|harem_, such that ephemeral water sources become increasingly widespread across the island during extreme mesic years (i.e., 2009), leading to decreased female dependency on permanent water, which in turn inhibits males from monopolizing all available water sources and females in an area. This situation would create equally attractive males with respect to water access during mesic summers, thereby dampening the rates of *I*_mares|harem_, as we observed at far distances from water. Conversely, during relatively dry summers, high water demands associated with gestation and lactation force females situated far from permanent water to frequently traverse far distances between forage and permanent or limited ephemeral water resources in an effort to minimize costs of breeding. Likewise, males farther from permanent water would be forced to balance energy expenditures associated with competitive interactions and acquisition of spatially distant resources, possibly reducing competitive differences among males. Hence, despite high costs to female horses that do not associate with strong dominant males (i.e., band instability; Kaseda, Khalil, & Ogawa, 1995, Linklater et al., 1999), long‐distance forays between limiting resources coincide with short‐term relationships and unstable bands that lead to what Linklater et al. (1999) referred to as “maverick mares” that exhibit a high propensity to disperse from home ranges situated far from permanent water during drought conditions. Thus, the demographic, environmental, and climatic effects we report here may be operating not only on individual resource requirements, but also through social behaviors to further influence opportunities for male selection.

Our post hoc analysis further revealed a plausible causal relationship between summer precipitation levels and the rate at which male mating inequality during mate acquisition changes with distance from water (Figure 5b). As characterized above, females are not expected to be choosy mates under extreme mesic or xeric meteorological conditions (Kokko & Monaghan, 2001) because summer precipitation determines availability of freshwater, and extreme dry or wet conditions can act on individual behaviors to swamp out male differences. For example, only during average years (e.g., 2008, 2011, and 2012), when a disproportionate availability of small, ephemeral water sources emerges across the island, did the effect of differences among males in their ability to monopolize these small ephemeral water sources and the local females that depend on them peak and reach an asymptote in *I*_mares|harem_ in relationship to summer precipitation (Figure 5b). This finding raises interesting questions regarding the potential for selection thresholds in relation to resource availability under the EPP hypothesis.

The effects of distance from water on mating inequality occurred irrespective of local density, in contrast to the suggestions of others who have investigated the effects of density on opportunity for sexual selection in other systems (see Kokko & Rankin, 2006). This may be attributed to our accounting for local ASR previously in our models, as ASR is considered the most important measure of density (relative density of the opposite sex) with regard to sexual selection (Kokko & Rankin, 2006). Despite no effect of total density, the density of unpaired males emerged as a strong predictor of selection associated with acquiring a harem, confirming the importance of unpaired males (Klug, Lindström et al., 2010). Links between unpaired males and opportunity for selection have been frequently proposed in the context of limiting resources (Ahnesjö et al., 2001; Klug, Lindström et al., 2010; Kokko & Rankin, 2006) and demonstrated to be important in fish (Klug, Heuschele et al., 2010), although this relationship has rarely been tested. Decreased density of unpaired males might coincide with lower total densities, decreased ASR in polygynous species, and decreased male–male interference, leaving proportionately fewer males unmated (Kokko & Rankin, 2006). Despite this, the underlying mechanism of unpaired males is complex because their local densities shift across space and time in response to tracking ephemeral water (and probably forage) resources under changing annual weather patterns. In the presence of annual fluctuations in summer precipitation, such environmental forces might explain the increased changes in opportunity for selection when acquiring harems among years that coincided with increases in opportunity for male selection.

This study increases our knowledge of the combined effects of demography, resources, and climate that shape the degree of male mating inequality in polygynous breeders. It provides a unique individual‐based perspective of local behavioral interactions and male mating success in a long‐lived species decomposed into hierarchical selection episodes across space and time. Our results highlight the significance of variable demographic and environmental conditions that change across fine‐grained local environments over a timescale shorter than the lifespan of an individual animal, and how these shape differential degrees of male mating inequality within the population. Our results raise interesting questions regarding the role of phenotypic plasticity in male mating strategies (Levins, 1968); although testing of spatially variable sexual selection is nascent, further work across species, mating systems, and environments will provide insights into selection beyond our current state of knowledge.

## CONFLICT OF INTEREST

The authors declare that they have no conflict of interest.

## AUTHOR CONTRIBUTIONS

JAM conceived the study, and JAM and PDM, along with additional laboratory members listed in the acknowledgments, collected, and processed data. JAM conceived and performed the analysis and wrote the manuscript with input from PDM. Both authors contributed substantially throughout all stages of manuscript preparation.

## ETHICAL APPROVAL

All sampling complied with the University of Saskatchewan Animal Care Protocol (20090032) and was approved by the Canadian Council on Animal Care, Canada Coast Guard, and Parks Canada Agency under Research License 14668.

## Data Availability

We will make data available from the Dryad Digital Repository: http://dx.doi.org/10.5061/dryad.20p4g44 (Manning & McLoughlin, 2019).
